# First case of evolved herbicide resistance in the holoparasite sunflower broomrape, *Orobanche cumana* Wallr.

**DOI:** 10.3389/fpls.2024.1420009

**Published:** 2024-06-04

**Authors:** Shiv Shankhar Kaundun, Alberto Martin-Sanz, Maribel Rodríguez, Tiberiu Serbanoiu, Jose Moreno, Eddie Mcindoe, Gael le Goupil

**Affiliations:** ^1^ Syngenta Ltd, Jealott’s Hill International Research Centre, Bracknell, Berkshire, United Kingdom; ^2^ Syngenta España S.A., Carmona-Lora de Rio, Sevilla, Spain; ^3^ Syngenta Agro SLR, Bucharest, Romania; ^4^ Syngenta Crop Protection AG, Werk Rosental, Basel, Switzerland

**Keywords:** sunflower broomrape, *Orobanche cumana*, virulence, imazamox, herbicide resistance

## Abstract

The development and commercialisation of sunflower varieties tolerant to acetolactate synthase (ALS)-inhibiting herbicides some 20 years ago provided farmers with an alternative method for the cost-effective control of *Orobanche cumana*. In 2020, however, two independent sunflower broomrape populations from Drama (GR-DRA) and Orestiada (GR-ORE), Greece, were reported to be heavily infested with *O. cumana* after application of the ALS-inhibiting herbicide imazamox. Here we have investigated the race of GR-DRA and GR-ORE and determined the basis of resistance to imazamox in the two Greek *O. cumana* samples. Using a set of five diagnostic sunflower varieties characterised by different resistant genes with respect to *O. cumana* infestation, we have clearly established that the GR-ORE and GR-DRA populations belong to the invasive broomrape races G and G+, respectively. Live underground tubercles and emerged shoots were identified at the recommended field rate of imazamox for GR-DRA and GR-ORE but not for two other standard sensitive populations in a whole plant dose response test using two different herbicide-tolerant sunflower hybrids as hosts. Sequencing of the ALS gene identified an alanine 205 to aspartate mutation in all GR-ORE samples. Most GR-DRA tubercles were characterised by a second serine 653 to asparagine ALS mutation whilst a few GR-DRA individuals contained the A205D mutation. Mutations at ALS codons 205 and 653 are known to impact on the binding and efficacy of imazamox and other imidazolinone herbicides. The knowledge generated here will be important for tracking and managing broomrape resistance to ALS-inhibiting herbicides in sunflower growing regions.

## Introduction

Sunflower broomrape, *Orobanche cumana* Wallr., is the most important biotic constraint to sunflower (*Helianthus annuus* L.) seed production outside North and South America (Molinero-Ruiz et al., 2015). It is an obligate holoparasite that infects the roots of sunflower early in the growing season and hinders the growth and development of the species by depleting the host of essential nutrients (Joel, 2009). At physiological maturity, one single broomrape plant can produce several tens of thousands of seeds which can remain viable for up to 20 years (Fernández-Martínez et al., 2015; Molinero-Ruiz et al., 2015). Sunflower seed losses caused by broomrape infestation can exceed 50% in susceptible *H. annuus* hybrids (Domínguez, 1996).

*O. cumana* is generally managed through breeding of genetic resistance into the host (Kaya, 2014). New sunflower varieties are regularly developed and released to manage *O. cumana* populations that have overcome current resistant traits (Molinero-Ruiz et al., 2015). Genetic resistance to *O. cumana* is in most cases qualitative (vertical) and controlled by major dominant genes, allowing classification of sunflower broomrape populations, so far, into eight races with increasing virulence from A to H (Vranceanu et al., 1981; Pérez-Vich et al., 2013; Martín-Sanz et al., 2016; Duca et al., 2022). The second way to control sunflower broomrape is via the use of herbicides on sunflower varieties that contain a resistant acetolactate synthase (ALS) target enzyme and some level of tolerance due to non-target-site based mechanisms (Kaya, 2015; Breccia et al., 2017). The first ALS-resistant sunflower was created via conventional breeding of a resistant trait from wild *Helianthus annuus* into elite cultivated sunflower varieties (Tan et al., 2005). The ALS-resistant wild sunflower population was discovered in Kansas in 1996 in a soybean field that was treated with imazethapyr for seven consecutive years (Al-Khatib et al., 1998). The wild Kansas *H. annuus* population was subsequently found to contain the alanine to valine mutation at ALS codon position 205 (Kolkman et al., 2004). The A205V target-site mutant sunflower, released as IMISUN or Clearfield variety, is resistant to imidazolinone subclass of ALS-inhibitors allowing in-crop use of herbicides such as imazamox for controlling broomrape and other important sunflower broadleaf and grass weeds (Mitkov et al., 2016). A second generation of herbicide-resistant sunflower (Clearfield Plus or CLHA-PLUS) was developed following EMS mutagenesis and selection with imazapyr (Sala et al., 2008a). CLHA-PLUS is characterized by the A122T mutation and shows increased tolerance to imidazolinones (IMI) herbicides compared to IMISUN and Clearfield sunflowers (Sala et al., 2008b; Pfenning et al., 2012). A third (SURES) and fourth (ExpressSun) class of sunflowers containing the P197L ALS resistant mutation was developed through introgression of the resistant allele from a wild *H. annuus* population and EMS mutagenesis, respectively (Miroslava, 2022). The SURES and ExpressSun varieties are tolerant to some sulfonylurea herbicides such as tribenuron-methyl but are sensitive to IMI herbicides. The use of IMISUN and CLHA-PLUS tolerant hybrids and IMI herbicides has proved very successful to control sunflower broomrape since their launch in 2003 and 2013, respectively (Molinero-Ruiz et al., 2015). As the technology also allows the simultaneous control of key non-parasitic weeds, their utilisation has exceeded 70% in some Mediterranean sunflower growing areas (Kaya, 2014).

During the last years highly aggressive broomrape populations that have overcome the existing resistance genes have been identified thus rendering the recalcitrant *O. cumana* populations prone to evolve resistance to IMI herbicides as well (Molinero-Ruiz et al., 2015; Martín-Sanz et al., 2016; Duca et al., 2022). Recently two Greek fields cultivated with the IMISUN and CLHA-PLUS sunflower hybrids were identified with severe attacks of *O. cumana* affecting more than 75% of the plants despite the use of imazamox. The objectives of this research therefore were to (a) assess the virulence of these two Greek sunflower broomrape populations using a set of diagnostic sunflower genotypes, (b) confirm imazamox resistance in the said populations under controlled conditions, and (c) identify any potential target-site resistance mechanism(s) to the imidazolinone herbicide.

## Materials and methods

### Orobanche cumana seed populations

Sunflower broomrape seeds were collected in 2020 from two Greek populations that have escaped a recommended application of imazamox (Pulsar^®^40, BASF). The populations were located at Drama (41°03’41.0”N 24°10’41.5”E) and Orestiada (41°39’44.0”N 26°13’19.2”E). The Drama and Orestiada broomrape seed populations are respectively referred to as GR-DRA and GR-ORE throughout the paper. An IMISUN hybrid was planted at Drama and several IMISUN and CLHA-PLUS hybrids were grown at Orestiada. A broomrape population from Seville province, Spain (SP-SE1) and another from Tulcea province Romania (RO-TU1) that are sensitive to imazamox were used as standards in herbicide resistance evaluations. Additionally, SP-SE1 and RO-TU1 which are both characterised as race G+ were used alongside three other broomrape populations from Seville province, Spain (SP-SE2), Haskovo province, Bulgaria (BU-HAS), and Tulcea province, Romania (RO-TU2) in race classification. SP-SE2, BU-HAS and RO-TU2 belong to races F, G and G+, respectively.

### Sunflower lines and hybrids

Five Syngenta commercial hybrids and one inbred line (P96) were used to assess the virulence of the two Greek *O. cumana* populations. The hybrids consisted of Hybrid 1, an old cultivar without any known resistance gene and susceptible to all evaluated broomrape populations according to Syngenta’s internal data; Hybrid 2, resistant to races A-E (*Or5* resistance gene); Hybrid 3, resistant to races A-F (*Or7*); Hybrid 4, resistant to races A-G (*Or5* and *Or7*); and Hybrid 5 (*Or5*, *Or7* and other unknown resistance genes), resistant to broomrape populations that are more aggressive than race G. The inbred line P96 is a genotype commonly used as a race F differential (Martín-Sanz et al., 2016).

The Sunflower Hybrid A (IMISUN) and Hybrid B (CLHA-PLUS) were used for the herbicide phenotypic evaluations. Both hybrids harbour the *Or5* resistance gene and are resistant to broomrape races A to E.

The different hybrids were selected based on extensive field trial data from several countries in addition to further tests performed in control glasshouse conditions. The hybrids were also evaluated against broomrape populations classified as race F_GV_, G_GV_ and G_TK_ in other studies (Martín-Sanz et al., 2016, Martín-Sanz et al., 2020; Fernández-Melero et al., 2023).

### Racial characterisation

The virulence of GR-DRA and GR-ORE was determined using known broomrape standards RO-TU1 (race G+), SP-SE1 (race G+), SP-SE2 (race F), BU-HAS (race G) and RO-TU2 (race G+), and five differential sunflower varieties (Hybrid 1, Hybrid 2, Hybrid 3, Hybrid 4 and Hybrid 5) and P96 line. Twelve plants of each sunflower line or hybrid were evaluated for each broomrape population. The experiments were conducted in 1 L pots containing a mixture of sand and peat (1:1 by volume). The soil was artificially contaminated with seeds from each broomrape population at a concentration of 20 ppm (20 mg seed kg^-1^ soil) and fertilised at a concentration of 6 gL^-1^ using Nutricote (Projar). The contaminated soil for each broomrape population was mixed to homogeneity in a cement mixer for 30 minutes. Sunflower seeds were germinated on moistened filter paper in Petri-dishes. Single emerged sunflower seedlings were transplanted in pre-prepared 1 L pots and placed in a growth chamber at 25/20°C (day/night) with a 16h photoperiod, photon flux density at 300 µmol m^-2^s^-1^ and irrigated as required. Phenotypic evaluation of virulence was determined 65 days after planting by calculating the incidence (percentage of sunflower plants infected by broomrape) and severity (average number of broomrape tubercles in the sunflower infected plants). Only broomrape tubercles with a differentiated stem were considered for estimation of incidence and severity.

### Evaluation of imazamox-resistance

Broomrape populations GR-DRA and GR-ORE were assessed for imazamox resistance alongside RO-TU1 and SP-SE1 which are known sensitive standards for the ALS-inhibiting herbicide. The four different populations were produced in the Hybrid B (CLHA-PLUS) as described above except for the pot volume which was 50 cm^3^. When the sunflower plants were at the four true leaf-stage (around 25 days after planting) they were sprayed with imazamox (Pulsar^®^40, BASF) using a Euro-Pulvé backpack sprayer, (model 68130) at a flow rate of 300 L ha^-1^. Sensitive standards SP-SE1 and RO-TU1 were treated with imazamox at 0, 0.39, 0.78, 1.56, 3.12, 6.25, 12.5, 25, 50 100 g ai ha^-1^ whilst the two suspected resistant populations GR-DRA and GR-ORE were tested at 0, 6.25, 12.5, 25, 50, 100, 200, 400, 800, 1600 g ai ha^-1^. Sixteen replicate pots were employed for each herbicide treatment (broomrape population X sunflower hybrid X herbicide dose) and 32 replicate pots were used for untreated controls. Thirty days after imazamox treatment the percentage of alive broomrape tubercles was recorded for each pot. This whole experiment for confirming broomrape resistance in the two Greek samples was replicated using the Hybrid A (IMISUN) as the sunflower host.

### Mechanism of imazamox resistance

The acetolactate synthase gene was sequenced to determine whether resistance to imazamox in the two Greek broomrape populations could be due to a target-site mutation. Thirty-two broomrape tubercles were analysed in all including five each from untreated RO-TU1 and SP-SE1 pots and 11 each from GR-DRA and GR-ORE collected from different imazamox treatments as shown in **Table 1**. Each individual *O. cumana* tubercle was chilled in liquid nitrogen and was pulverised into a fine, homogeneous powder using mortar and pestle. Total genomic DNA was extracted using Qiagen’s DNeasy Plant Mini Kit (Cat. No. 69104, Qiagen, Germany) following the manufacturer’s instructions. DNA integrity was checked on a gel and quantified with Nanodrop. The quasi-complete ALS gene was amplified by PCR using the forward 5’ CACCGTCGACAATGGCGG 3’ and reverse 5’ ATCACATCCTTAAACGCACC 3’ primer pairs derived from *Orobanche minor*, the closest species for which the ALS gene was available (GenBank reference: IADW01087971.1). NEB Q5 Polymerase (Cat. No. M0491S, NEB, MA, USA) was employed to amplify the high GC content of the *Orobanche minor* ALS gene. PCRs were performed on an Eppendorf Master Cycle Gradient Thermocycler Model 96 programmed for an initial denaturation step of 98°C for 1 min, followed by 30 cycles of 30 s at 98°C, 10 s at 64°C and 1 min at 72°C. A final extension step for 2 min at 72°C was also included. PCR product was checked on a gel and column-purified with Invitrogen’s PureLink^®^ PCR Purification Kit (Cat. No K310001, Invitrogen, MA, USA) and sent for Sanger sequencing at Genwiz. The PCR fragment was fully sequenced using four forward and two reverse internal sequencing primers.

**Table 1:** Origin and genotypes at ALS codons 205 and 653 of 32 tubercles collected at different imazamox rates.

Tubercle	Population	Imazamox rate g ai ha^-1^	Genotype at codon 205	Genotype at codon 653
1	SP-SE1	0	AA205	SS653
2	SP- SE1	0	AA205	SS653
3	SP- SE1	0	AA205	SS653
4	SP- SE1	0	AA205	SS653
5	SP- SE1	0	AA205	SS653
6	RO-TU1	0	AA205	SS653
7	RO-TU1	0	AA205	SS653
8	RO-TU1	0	AA205	SS653
9	RO-TU1	0	AA205	SS653
10	RO-TU1	0	AA205	SS653
11	GR-DRA	0	AA205	NN653
12	GR-DRA	0	AA205	NN653
13	GR-DRA	0	AA205	NN653
14	GR-DRA	0	AA205	NN653
15	GR-DRA	0	AA205	NN653
16	GR-DRA	50	AA205	NN653
17	GR-DRA	50	AA205	NN653
18	GR-DRA	50	AA205	NN653
19	GR-DRA	100	AA205	NN653
20	GR-DRA	200	DD205	SS653
21	GR-DRA	200	DD205	SS653
22	GR-ORE	0	DD205	SS653
23	GR-ORE	0	DD205	SS653
24	GR-ORE	0	DD205	SS653
25	GR-ORE	0	DD205	SS653
26	GR-ORE	0	DD205	SS653
28	GR-ORE	50	DD205	SS653
29	GR-ORE	100	DD205	SS653
31	GR-ORE	100	DD205	SS653
27	GR-ORE	200	DD205	SS653
30	GR-ORE	400	DD205	SS653
32	GR-ORE	800	DD205	SS653

## Results

### Virulence of the two Greek broomrape populations

The five reference broomrape populations employed in the racial characterisation study behaved as expected. The reference populations, resistant to race F or above, were able to attach to the sunflower roots and develop a differentiated stem in every susceptible Hybrid 1 and race A-E resistant Hybrid 2 plant (**Table 2**). The average number of broomrape tubercles observed in Hybrid 1 ranged from 4.3 for RO-TU2 to as many as 21.3 for SP-SE2 (**Table 3**). A respectively low and high number of tubercles were also detected for RO-TU2 (5.8) and SP-SE2 (20.8) in Hybrid 2. Consistent with its race F status, SP-SE2 could not infest Hybrid 3, Hybrid 4, Hybrid 5 and P96 which are resistant to races F or above. BU-HAS, a race G broomrape population, was able to colonise Hybrid 3 and P96. RO-TU2 (race G+) parasitised all sunflower varieties except Hybrid 5 which is resistant to broomrapes that are more aggressive than race G. RO-TU1 parasitised all sunflower genotypes consistent with its race G+ status. SP-SE1, in agreement with its distinct G+ profile, could infest all sunflower genotypes except P96. The Greek population GR-ORE being investigated parasitised Hybrid 1, Hybrid 2, the race F-resistant Hybrid 3 and P96 sunflowers but not Hybrid 4 and Hybrid 5 hybrids which are resistant to race G broomrapes. The average number of GR-ORE tubercles was comparatively low at 2.7 in Hybrid 3 but as high as 21.2 in P96 (**Table 3**). GR-ORE was thus classified as a race G broomrape. With its ability to parasitise all but the sunflower Hybrid 5, the Greek population GR-DRA was assigned to race G+. An average of 5.3 GR-DRA tubercles was recorded in Hybrid 4 (resistant to race G) and 26 in P96 (resistant to race F).

**Table 2:** Percentage of susceptible sunflower plants in six sunflower hybrids and lines (incidence).

GENOTYPE	SP-SE2	BU-HAS	RO-TU2	SP-SE1	RO-TU1	GR-ORE	GR-DRA
Hybrid 1	100.0	100.0	100.0	100.0	100.0	100.0	100.0
Hybrid 2	100.0	100.0	100.0	100.0	100.0	100.0	100.0
Hybrid 3	0.0	83.3	100.0	100.0	100.0	66.7	100.0
Hybrid 4	0.0	0.0	50.0	100.0	100.0	0.0	100.0
Hybrid 5	0.0	0.0	0.0	100.0	100.0	0.0	0.0
P96	0.0	100.0	100.0	0.0	66.7	100.0	100.0
RACE	F	G	G+	G+	G+	G	G+

**Table 3:** Average number of *O. cumana* attachments with a differentiated stem in six sunflower hybrids and lines (severity).

GENOTYPE	SP-SE2	BU-HAS	RO-TU2	SP-SE1	RO-TU1	GR-ORE	GR-DRA
Hybrid 1	21.3 ± 1.0	16.8 ± 1.0	4.3 ± 0.7	20.2 ± 1.3	10.3 ± 0.9	17.0 ± 1.6	14.6 ± 1.5
Hybrid 2	20.8 ± 1.4	19.3 ± 1.2	5.8 ± 0.7	16.0 ± 0.9	14.7 ± 1.4	14.2 ± 1.0	18.4 ± 1.5
Hybrid 3	0.0	6.7 ± 1.5	12.0 ± 0.9	20.4 ± 1.4	13.0 ± 0.8	2.7 ± 0.8	12.8 ± 1.3
Hybrid 4	0.0	0.0	3.5 ± 1.4	8.8 ± 0.9	10.3 ± 0.7	0.0	5.3 ± 0.4
Hybrid 5	0.0	0.0	0.0	6.3 ± 0.6	9.5 ± 0.8	0.0	0.0
P96	0.0	5.0 ± 0.6	7.0 ± 1.0	0.0	1.4 ± 0.4	21.2 ± 1.4	26.0 ± 1.3
RACE	F	G	G+	G+	G+	G	G+

### Investigation of imazamox resistance in the two Greek *Orobanche* populations

**Figure 1:**
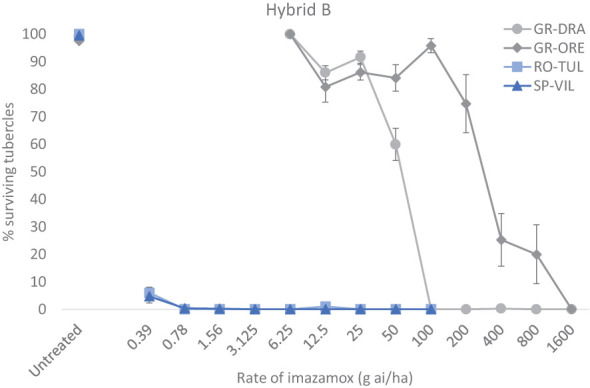
Responses of four broomrape populations to imazamox in sunflower Hybrid B.

**Figure 2:**
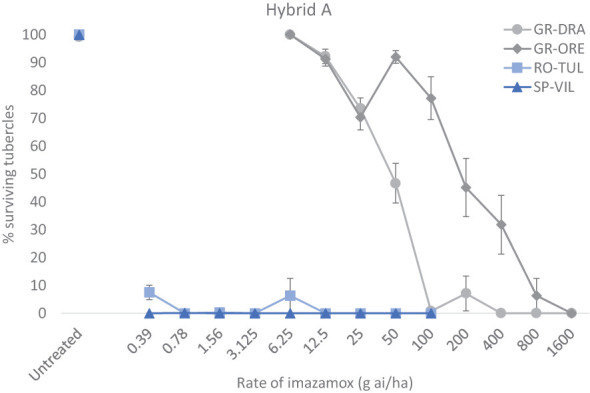
Responses of four broomrape populations to imazamox in sunflower Hybrid A.

The two standard sensitive populations SP-SE1 (race G+) and RO-TU1 (race G+) and two GR-DRA (race G+) and GR-ORE (race G) populations being investigated adequately parasitised the Hybrid A and Hybrid B hosts allowing determination of imazamox resistance in the two Greek samples. However, there was a relatively large variation in the total number of tubercles among replicate pots for all four populations and herbicide rates tested. On average, the total number of broomrape tubercles was around 50-65 for GR-ORE, GR-DRA and SP-SE1 and half as much for RO-TU1 in untreated sunflower pots. The percentage of live tubercles decreased rapidly as the rate of imazamox increased, especially for the two sensitive broomrape populations in both sunflower hybrids (**Figures 1**, **2**). In Hybrid B for example, only 4.7% of SP-SE1 and 6.0% of RO-TU1 were recorded at the lowest rate of 0.39 g ai ha^-1^ of imazamox tested (**Figure 1**). From 0.78 g ai/ha imazamox onwards no live RO-TU1 tubercles were observed whilst only 2.4% of live SP-SE1 tubercles were detected at the second rate of the IMI herbicide. A few live SP-SE1 individuals were still observed at 1.56 and 3.3 g imazamox rates but these only amounted to 0.3 and 0.2% of the total number of tubercles. In contrast, all GR-DRA and GR-ORE tubercles were alive at 6.5 g of imazamox. At the recommended field rate of 50 g ai ha^-1^ imazamox, 59.9% of GR-ORE and 84% of GR-DRA tubercles survived the herbicide treatment. No GR-DRA survivors were found beyond 100 g ai ha^-1^ imazamox whilst as much as 20% of GR-ORE live tubercles was recorded at 800 g ai ha^-1^ of the herbicide. The broomrape resistance profiles in Hybrid A were comparable with those in the Hybrid B with the two sensitive standard populations being killed at relative low rates of imazamox (**Figure 2**). In Hybrid A, GR-DRA survivors were identified with up to 50 g ai ha^-1^ imazamox but not beyond and live GR-ORE tubercles were present up to 800 g ai ha^-1^ of the IMI herbicide. The large difference in herbicide response between GR-ORE and GR-DRA on the one side, and the two known susceptible SP-SE1 and RO-TU1 populations on the other side, confirmed imazamox resistance in the two Greek populations. The relatively higher level of imazamox resistance in GR-ORE as compared to GR-DRA suggests that two different mechanisms may be at play.

### Mechanism of imazamox resistance

Thirty-two individual broomrape tubercles collected from untreated and treated GR-DRA, GR-ORE, SP-SE1 and RO-TU1 were sequenced to determine whether a target-site mutation was responsible for imazamox resistance in the two Greek populations (**Table 1**). Polymerase chain reactions generated a 1945 base pair fragment that covered 98% of the acetolactate synthase gene. The amplified fragment encompassed 11 bases of the 5’ UTR and the first base of codon 645 of *ALS*. Analysis of the *ALS* sequences from *O. cumana* showed on average 67% and 75% homologies at the nucleotide and protein levels respectively, with publicly available ALS from *O. minor.* The ALS gene was very conserved among all 32 *O. cumana* samples, except for a cytosine to an adenine transversion at the second bases of codons 205 and 653 (Arabidopsis equivalent), resulting in an alanine 205 to aspartate mutation and a serine 653 to asparagine mutation, respectively. The A205D mutation was present at the homozygous state in all untreated and treated GR-ORE samples and in two GR-DRA tubercules that have survived a high rate (200 g ai ha-1) of imazamox treatment (**Table 1**). The eight GR-DRA tubercles collected from untreated pots or at a relatively lower rate of imazamox were homozygous for the S653N mutation. No A205D and S653N double mutants were identified among the GR-DRA or GR-ORE samples. The nucleotide sequences of all 10 tubercules from the standard sensitive SP-SE1 and RO-TU1 populations were identical and characterised by homozygous wild type AA205 and SS653 alleles.

## Discussion

### Racial characterisation of the two Greek *O. cumana* populations

In Southeast Europe, *O. cumana* has become an increasingly troublesome species affecting the yield of sunflower (Molinero-Ruiz et al., 2015; Rubiales, 2020). Investigation of broomrape virulence from sunflower growing countries is important to guide the breeding and selection of tolerant varieties for effectively managing *O. cumana* (Perez-de-Luque et al., 2009; Shi and Zhao, 2020). Over the years, several surveys have been conducted from broomrape samples collected from Spain, Turkey, Romania, Bulgaria, Serbia and Russia (Malek et al., 2017; Škorić et al., 2021). Here, the racial evaluation of the two Greek populations to a set of differential sunflower host genotypes showed that the population from Drama belonged to race G+ while the other population from Orestiada belonged to race G. The identification of highly virulent broomrape strains from Greece is in line with a recent investigation which showed that the two races were prevalent in most South-eastern European countries except for Serbia whereby the strains were found to belong to race E or were less virulent than E (Duca et al., 2022). It is noteworthy that the race G strain identified in the Drama population differed from some Spanish race G populations which could not infest P96, a key race F differential. The distinct Spanish race G strain was baptised race G_GV_ (Martín-Sanz et al., 2016). The virulence identified in the Greek populations in part explicate why they could infest the race A-E resistant sunflower hybrids planted at Drama and Orestiada. The appearance of increasingly virulent populations in recent years could be explained by the intensity of sunflower breeding for resistance to new strains of broomrape, thereby exerting immense selection pressure on the parasite to evolve more virulent races (Molinero-Ruiz et al., 2015; Miraille et al., 2022).

### Evolution of imazamox resistance in *O. cumana*

Our detailed study here confirmed high levels of imazamox resistance in the two Greek populations. This is the first instance of herbicide resistance identified in sunflower broomrape and the second case of resistance evolution in a holoparasite. The first occurrence of herbicide failure in a holoparasite was in an Israeli field dodder, *Cuscuta campestris*, population that was also found to be resistant to ALS-inhibiting herbicides (Rubin, 1995).

Resistance to ALS herbicides is by far the most common among all other modes of action with as many as 172 different species, including *O. cumana*, being affected (Powles and Yu, 2010; Heap, 2023). The rapid and widespread evolution of resistance to ALS herbicides is attributed to the large number of ALS-inhibiting herbicides developed and commercialised over the last 40 years and their intensive use in all major crops and growing regions worldwide (Zhou et al., 2007; Mendes et al., 2022). ALS herbicides are potent inhibitors, often with long residual activity, providing effective kill of susceptible individuals, thus exerting high selection pressure on weed populations to evolve resistance (Whitcomb, 1999; Zhou et al., 2007). The intrinsic prevalence of ALS resistant genes in untreated weed populations is much higher than with other modes of action and several mutations that endow target-site resistance have little to no impact on plant fitness due to the non-competitive inhibition of the ALS enzyme (Powles and Yu, 2010; Gaines et al., 2020). In highly outcrossing and heterogeneous species, such as *Alopecurus myosuroides* and *Lolium rigidum* resistance was identified within a few years of ALS herbicide application (Heap, 2023). Compared to most other species, resistance evolution to ALS herbicides in *O. cumana* has been relatively slow mainly because chemical control is generally combined with sunflower resistance genes, including *Or_SII_* and *Or_DEB2_* that impact on the attachment and development of the holoparasite (Martín-Sanz et al., 2020; Fernández-Aparicio et al., 2022). This double system of protection explains why the vast majority of *O. cumana* populations is still effectively controlled with ALS herbicides even after 20 years of use. Other contributing factors for the slow evolution of resistance include the relatively lower selection pressure exerted on *O. cumana* by ALS herbicides. Sunflower broomrape is a non-photosynthetic root parasite plant that relies entirely on the host for its nutrients and water (Fernández-Martínez et al., 2015). Chemical control of *O. cumana* is complicated because it produces a large number of seeds that can survive in the soil for several years and sprout when stimulants from the host roots are detected (Plaza et al., 2004; Wu et al., 2022). For effective control of *O. cumana*, the herbicide must be sprayed at the right growth stage of the sunflower plant so it can be optimally translocated to the host roots and suppress the developing broomrape (Joel et al., 2007). The level of *O. cumana* control is also very much dependent on the number and depth of the holoparasite attachment (Eizenberg et al., 2009). Altogether, the level of kill and selection pressure from the application of ALS herbicides on *O. cumana* is not as high as with other non-parasitic weeds due to escapes of susceptible individuals that ultimately contribute to the *O. cumana* seed bank. Additionally, sunflower is very rarely grown in the same field year after year in Southeast Europe. Instead, the oilseed crop is often rotated with corn, cotton, winter oilseed rape and small grain cereal crops. The relatively long crop rotation and deep cultivation often practiced may explain the slow evolution of resistance to imazamox in sunflower broomrape, and also why only a few cases of ALS resistance have been recorded in other key sunflower weeds such as *Ambrosia artemisiifolia* and *Amaranthus retroflexus* in Southeast Europe (Heap, 2023).

### Mechanism of imazamox resistance in the Greek broomrape populations

An alanine 205 to aspartate amino acid change was found to be present in all samples from the imazamox resistant population from Orestiada. A few individuals of the population from Drama also contained the A205D mutation whilst most samples were characterised by the S653N mutation. The role of the S653N mutation in conferring resistance to IMI herbicides has clearly been established in several published studies (Powles and Yu, 2010). The heterologous expression of the Arabidopsis ALS gene bearing the wild serine and mutant asparagine alleles in *E. coli* and inhibition assays of the corresponding proteins showed that the S653N endowed high levels of resistance to IMIs whilst being mostly susceptible to sulfonylurea herbicides (Duggleby et al., 2008). The S653N mutation has also been documented in several weed species that have evolved resistance to ALS herbicides (Heap, 2023). In *Amaranthus tuberculatus* for example, whole plant dose response tests showed that the S653N mutation conferred resistance to imazethapyr but was susceptible to thifensulfuron (Patzoldt and Tranel, 2007). The S653N ALS mutation is the basis of resistance in some IMI-tolerant crops (Tan et al., 2005). ALS-tolerant Clearfield wheat bearing the S653N mutation, for instance, is resistant to imazamox but sensitive to tribenuron-methyl. In contrast, the importance of the A205D mutation in conferring resistance to ALS-inhibiting herbicides has not been fully investigated so far. The only occurrence of this mutation was in a single plant of an *A. palmeri* population from Spain (Barco-Antoñanzas et al., 2022). The A205D mutation was present as a double mutant alongside the W574L mutation that is known to confer broad resistance to all ALS-inhibiting herbicides. Therefore, the exact role of the A205D mutation in conferring resistance to ALS herbicides could not be assessed adequately. However, wild type alanine 205 is known to be a key determinant in the binding of some ALS inhibiting herbicides since two mutations at this codon position were found to affect the efficacy of some ALS-inhibitors (Heap, 2023). A *Conyza canadensis* population carrying the A205V mutation was found to be highly resistant to pyrithiobac-sodium (pyrimidinyl-thiobenzoate) and imazapyr (imidazolinone) and moderately resistant to sulfometuron-methyl (sulfonylurea) (Matzrafi et al., 2015). The A205V mutation is the basis of tolerance to imazamox in IMISUN sunflower indicating that the A205D mutation may also be conferring resistance to imazamox to *O. cumana* (Sala et al., 2012). Similarly, the Ala-205-Phe substitution identified for the first time in a *Poa annua* population, expressed in *E. coli* and assayed *in vitro* with a range of ALS herbicides was found to be resistant to all five ALS subclasses of herbicides except for florasulam (Brosnan et al., 2016). When present in the same *A. palmeri* population and genetic background, the S653N change endowed higher levels of resistance to the IMI herbicide imazethapyr compared to the A205V mutation (Palmieri et al., 2022). Here, it appears that the A205D mutation confers higher levels of resistance than the S653N mutation as a larger shift in imazamox efficacy was identified in the A205D mutant Orestiada population versus the Drama population which is essentially S653N mutant with some individuals containing the A205D mutation. This is not surprising given that resistance to ALS-inhibiting herbicides tends to be compound and mutation specific (Powles and Yu, 2010; Gaines et al., 2020).

### Detection of herbicide resistance in broomrape

Given the importance of ALS herbicides for the control of *O. cumana*, it is imperative to determine whether resistance is also present in other sunflower regions and countries where the holoparasite is prevalent. Here, resistance to imazamox was confirmed using the time consuming, space-inefficient and laborious classical whole plant pot assay (Burgos et al., 2013). Furthermore, many pots were required for resistance confirmation as the number of tubercles among replications was appreciably variable even for untreated samples. The need for a sensitive sunflower host that can be effectively parasitised by *O. cumana* adds another level of difficulty in herbicide resistance investigations. As with some other weed species, sunflower broomrape tested under controlled glasshouse conditions was found to be very sensitive to imazamox with the susceptible populations being killed in some instances with as low as 0.39 g ai ha^-1^. Thus, using a single recommended rate of 50 g ai ha^-1^ could cause over-kill and lead to false negatives of resistance especially for populations characterised by target-site mutations or other mechanisms that provide lower levels of resistance than the A205D or S653N amino acid changes. The classical whole plant pot assay with a wide range of herbicide rates and replicate pots cannot be sustainably employed if large numbers of broomrape populations need to be tested. Instead, a Petri-dish seed-based assay could be envisaged using proven sunflower broomrape germination stimulants such as GR24 and a wide range of pre-determined imazamox rates (Lachia et al., 2014; Chen et al., 2021). If successful, minimal efforts and space would be required, and test results would be available in less than half the time required for a whole plant pot test. The seed assay will also facilitate the determination of cross-resistance endowed by different ALS resistance mechanisms as a sunflower host with specific herbicide tolerances would no longer be required. Alternatively, if resistance is found to be mainly due to known target-site mutations, high throughput DNA-based molecular assays could be developed for the quick and efficient determination of resistance in large number of populations (Kaundun et al., 2019). This will be relatively straightforward and highly applicable across populations given the extreme conservation of the ALS gene among *O. cumana* populations and individuals.

### Sustainable use of ALS herbicides for controlling *O. cumana*

Chemical control of *O. cumana* in sunflower is currently limited to a few imidazolinone herbicides (Kolkman et al., 2004). Combined with sunflower resistant genes which were regularly discovered and bred into elite sunflower varieties, herbicide use has considerably improved management of *O. cumana* in Southeastern Europe for the best part of the last two decades (Fernández-Martínez et al., 2012; Molinero-Ruiz et al., 2015). The appearance of progressively more virulent populations has significantly increased the risk of resistance evolution to ALS herbicides in *O. cumana*. Therefore, perpetual breeding and selection efforts are required to keep pace with the development and spread of new sunflower broomrape races. Recent progress in sunflower genomics provides additional tools for plant breeders to find durable solutions against broomrape spread and virulence in sunflower (Cvejić et al., 2020). The discovery and breeding of multiple resistant genes via a pyramiding approach will drastically decrease the risk of evolution of virulent broomrape races (Velasco et al., 2016; Miraille et al., 2022). For the long-term management of the holoparasite, it is imperative to adopt an integrated approach combining chemical and genetic control with sound agronomic practices, including rotation with trap crops, use of clean seeds and measures to limit seed spread via agricultural machinery and tillage tools (Rubiales et al., 2009; Habimana et al., 2014). The construction of a computer-based model of *O. cumana* that accounts for the full life cycle of the species and diverse control methods will help design useful strategies for the sustainable management of sunflower broomrape.

## Data availability statement

The datasets presented in this study can be found in online repositories. The names of the repository/repositories and accession number(s) can be found below: https://www.ncbi.nlm.nih.gov/genbank/, accession number ID is 2803848.

## Author contributions

SK: Conceptualization, Investigation, Methodology, Supervision, Writing – original draft, Writing – review & editing. AM: Conceptualization, Investigation, Methodology, Project administration, Resources, Supervision, Writing – original draft. MR: Investigation, Methodology, Writing – review & editing. TS: Investigation, Resources, Writing – review & editing. JM: Investigation, Methodology, Writing – review & editing. EM: Formal analysis, Investigation, Writing – review & editing. GL: Conceptualization, Methodology, Writing – review & editing.
